# Measurement of mass force field driving water refilling of cuttage

**DOI:** 10.1038/s41598-024-59716-x

**Published:** 2024-04-18

**Authors:** Mingwei Xu, Ke Li, Yanling Xue, Feixiang Wang, Zhixuan Liu, Tiqiao Xiao

**Affiliations:** 1 Research Center for Shanghai Synchrotron Radiation Facility, Shanghai Advanced Research Institute, Chinese Academy of Sciences, Shanghai, 201204 China; 2 Shanghai Institute of Applied Physics, Chinese Academy of Sciences, Shanghai, 201800 China; 3 University of Chinese Academy of Sciences, Beijing, 100049 China; 4 Hunan Rice Research Institute, Hunan Academy of Agricultural Sciences, Changsha, 410125 China

**Keywords:** X-rays, Plant physiology

## Abstract

Cuttage is a common plant cultivation method, and the key to its survival is the restoration of water refilling, which remains unclear up to now. We report 3D dynamic imaging of water refilling of cuttage without resorting to any contrast agent. Hydrodynamics of the refilled water flow over time reveals the existence of a unit mass force field with a gradient along the refilling direction, which means that cutting plants also have a gradient force field to drive the recovery of water refilling, as predicted by Cohesion-Tension theory in normal plants. We found that force fields of different functional regions are isolated and independently distributed, which is conducive to ensure the safety of water transmission. At the same time, we also found that there is a so-called "inchworm effect" in the mass force field, which contributes to the force transfer inside the cutting through local force accumulation. Results of this paper demonstrate that the developed method for the measurement of mass force field in-vivo is applicable to help decipher the mechanism of plant water refilling.

## Introduction

Water plays a vital role in the process of plant photosynthesis, temperature regulation and cell metabolism. Plants carry water from soil to leaves several meters or even a hundred of meters high without the help of mechanical pumps. This anti-gravity water delivery mechanism has been a puzzle for botanists and physicists^1^. According to hydrodynamic principles, there is a mass force field within the plant, driving water from bottom to top^2,3^. So far, Cohesion-Tension theory (CTT) has been the mainstream explanation of plant water transport mechanism^4–6^. CTT pointed out that the tensile force generated by leaf transpiration and the cohesion between water molecules in the continuous liquid column contributed to the plant’s water transport. As a common plant propagation method, cuttage has important economic value. When a stem segment is cut before cuttage, the continuous distribution of liquid column from roots to leaves is cut off^7^, meanwhile the transpiration’s tensile force of leaves disappears. This seems to suggest that the cutting does not satisfy apparently the two basic premises of the CTT theory. According to the Scholander assumption, water will be discharged from the vessels by transpiration or gravity when transpiring plants are cut in air, regardless of the relationship between the pressure inside the plants and the atmospheric pressure, resulting in cavitation^8–13^. What’s more, Torres-Ruiz et al*.*^14^ found that cutting plants under tension in the air can exacerbate the degree of xylem embolism. Sperry and Tyree investigated the hypothesis that water-stress induced xylem embolism is caused by air aspirated from embolized vessels (e.g. embolized by physical damage) into functional vessels through pores in the inter-vessel pit membranes, and they conducted experiments to prove it^12^. Cavitation and refilling cycles are a normal occurrence in naturally growing plants due to factors such as freeze–thaw cycles and water stress^15–17^. As plants are cut in the air before during cuttage, cavitation spreads in the cuttings. Therefore, refilling is a vital factor in the success of the cuttage as it ensures the resupply of water^18^. Although several studies have suggested that root pressure^19^, the disjoining pressure concept for liquid thin-flms^20^ and solute osmotic pressure^21^ are probably the primary mechanisms for plant refilling, the mechanism of water refilling dynamics remains unclear due to the lack of effective in-situ testing methods^22^.

A lot of theoretical and experimental studies have been carried out on the hydrodynamic mechanism of water transmission in plants. In 1948, Van den Honert compared the hydraulic resistance of plant tissue with the resistance in Ohm’s law and laid the foundation for quantitative analysis of plant water transport mechanism to a certain extent^23,24^. Experimental exploration has also been carried out by researchers with the help of artificial trees^25,26^, dyeing^27,28^, heat tracking^29–31^, pressure measuring^32–34^, D_2_O tracer^35,36^ etc. X-ray phase-contrast imaging was employed to track the change of gas–water interface and a model of embolism repair was provided ^21,37^. With the combination of MRI and micro-CT, the influence of transverse pressure gradient in the xylem on water transport was revealed^38^. However, methods available is confronted with difficulties in revealing the water flow three dimensionally with simultaneous high spatial and temporal resolution. The X-ray dynamic micro-CT is capable of 3D in-situ dynamic imaging and has been successfully used to study the dynamic evolution of 3D microstructure of yellow mealworm^39^, insect respiratory^40^, microwave ceramic sintering^41^ and living ant^42^. Due to the complex vascular bundles and tissues in plant stems, and the minute density difference between living plant tissues and water, conventional dynamic micro-CT is powerless to reveal hydrodynamics of plant cuttage.

With the benefit of move contrast imaging^43–46^, Li et al.^47^ found that water refilling along maize leaf vessel first infiltrated along the vessel wall and then delivered rapidly. Further combining move contrast imaging with dynamic micro-CT to realize 3D in-situ imaging of the water refilling process at the initial stage of willow cuttage, Xu et al.^48^ revealed three typical modalities of refilling along the vessel and found the relay refilling mode based on the three-dimensional spatial structure relationship. In this paper, we developed a method based on move contrast microtomography to measure in-vivo the mass force field for revealing the hydrodynamic mechanism driving water refilling of plant cuttage.

## Results

### Occurrence of water refilling

**Figure 1:**
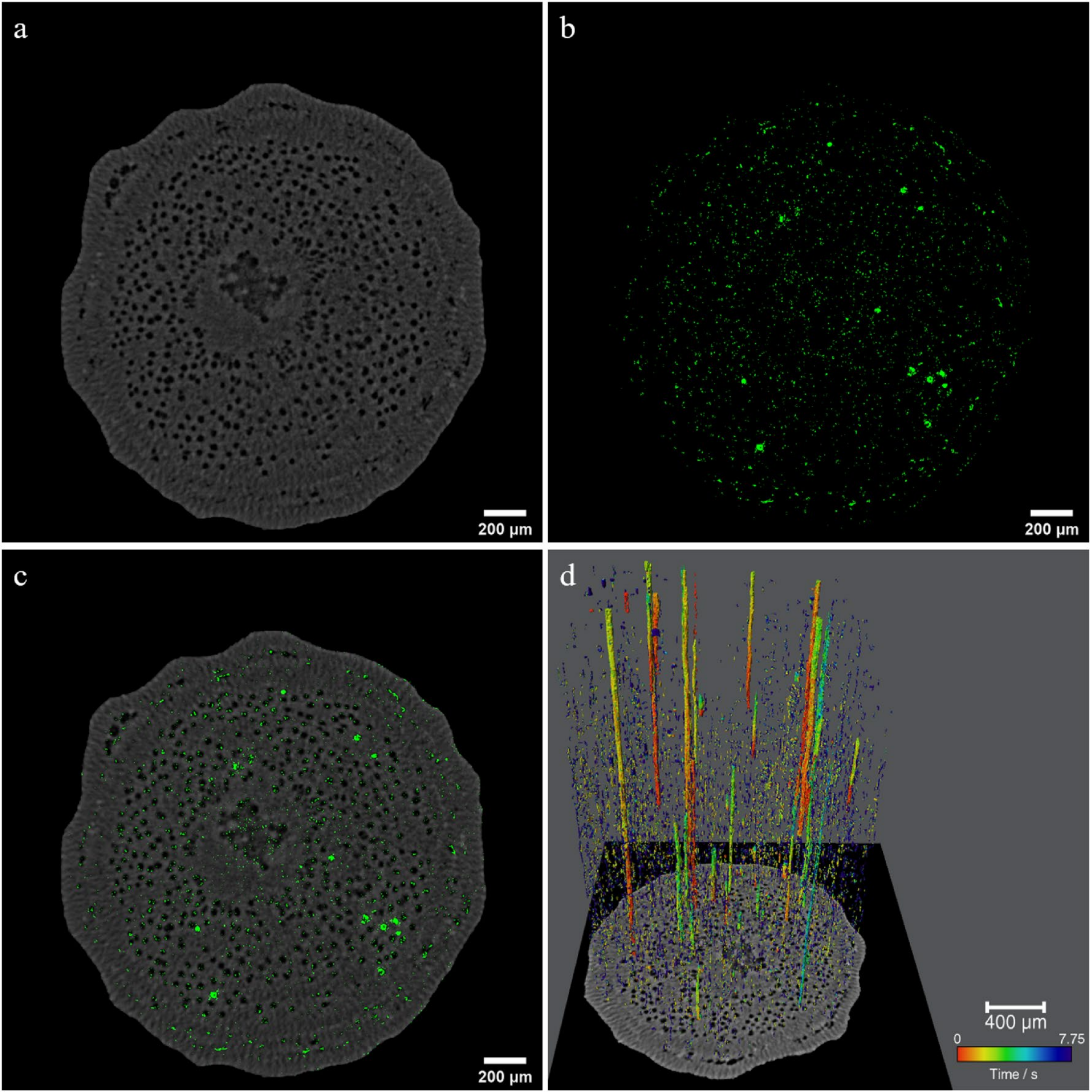
MCXCT results for willow branch water refilling. (**a**) CT slice No. 500 of the willow branch. (**b**) MCXCT slice No. 500 at 7.75 s. (**c**) Fusion of panels (**a**) and (**b**). (**d**) 3D dynamic spatiotemporal distribution of willow branch water refilling obtained from MCXCT.

To reveal the hydrodynamics of refilling, we need to obtain the refilling information on the location, volume, flow rate, and their evolution with time in order. According to the principle of move contrast CT, it is the best means to acquire the related information. The internal 3D microstructure of the willow branch and the evolution of the water refilling trajectory with time were acquired through in-line phase contrast X-ray CT (PCXCT) and move contrast CT (MCXCT) simultaneously. As a result, we are able to understand the time evolution of refilling and the relationship between refilling and the structure of the willow branch. Figure 1 shows the four-dimensional spatial and temporal distribution of water refilling in the willow branch at the initial stage of cuttage. Figure 1a shows the 500th slice of PCXCT. There are many intricate black circular holes in the slice, which are the distribution of the vessels in the cross section. Figure 1b shows the 500th slice of MCXCT. The green part represents the amplitude signal of the move contrast, which is the distribution of refilled water. The refilled water is distributed discretely in most regions of the cross section and accumulates in some regions.

Since MCXCT and PCXCT use the same data for reconstruction with fully consistent projections, the spatial positions of each pixel on the PCXCT and MCXCT slices are in one-to-one correspondence. The distribution of refilling in the willow branch can be obtained by fusing the two data sets, as shown in Fig. 1c. The fusion results show that refilling accumulates significantly in some vessels, indicating that refilling is active in these parts. Some of the inner walls of the vessels are scattered with green dots, indicating that these vessels are experiencing water infiltration from the tissues to the vessels. The tissues outside the vessel also scattered a large amount of refilling signals, indicating that the tissue also participated in the refilling process. The amplitude and phase of the move contrast represent the refilling trajectory and its evolution with time, therefore combining amplitude diagram with phase diagram to obtain the evolution of refilling with time in 3D space (Fig. 1d). The supplementary video (water refilling-3D dynamic.mp4) shows the temporal evolution of refilling in three-dimensional space. It shows that the refilling of long vessels mainly occurs from bottom to top, but the starting height of refilling varies with different vessels. This suggests that the refilling process of water is not a direct delivery along a single vessel from proximal to distal, but a relay delivery mode that couples the vessels through the tissues. In Supplementary Fig. S3, it is found that the water in the vessel will accumulate and fill the vessel, forming a liquid column from bottom to top. This further suggests that refilling within a vessel comes from tissue infiltration around the vessel. In addition to the apparent vessel delivery, many dotted water penetration signals distributed over the entire 3D microscopic field of view of the willow branch can also be observed. These refilling signals are distributed in the surrounding tissues of the vessels and in the cavitated vessels during the initial stages of refilling. From the color distribution of the dotted signal, the refilling process occurs from proximal to distal, which is consistent with the existing knowledge^48^.

### Time evolution of refilled water volume

**Figure 2:**
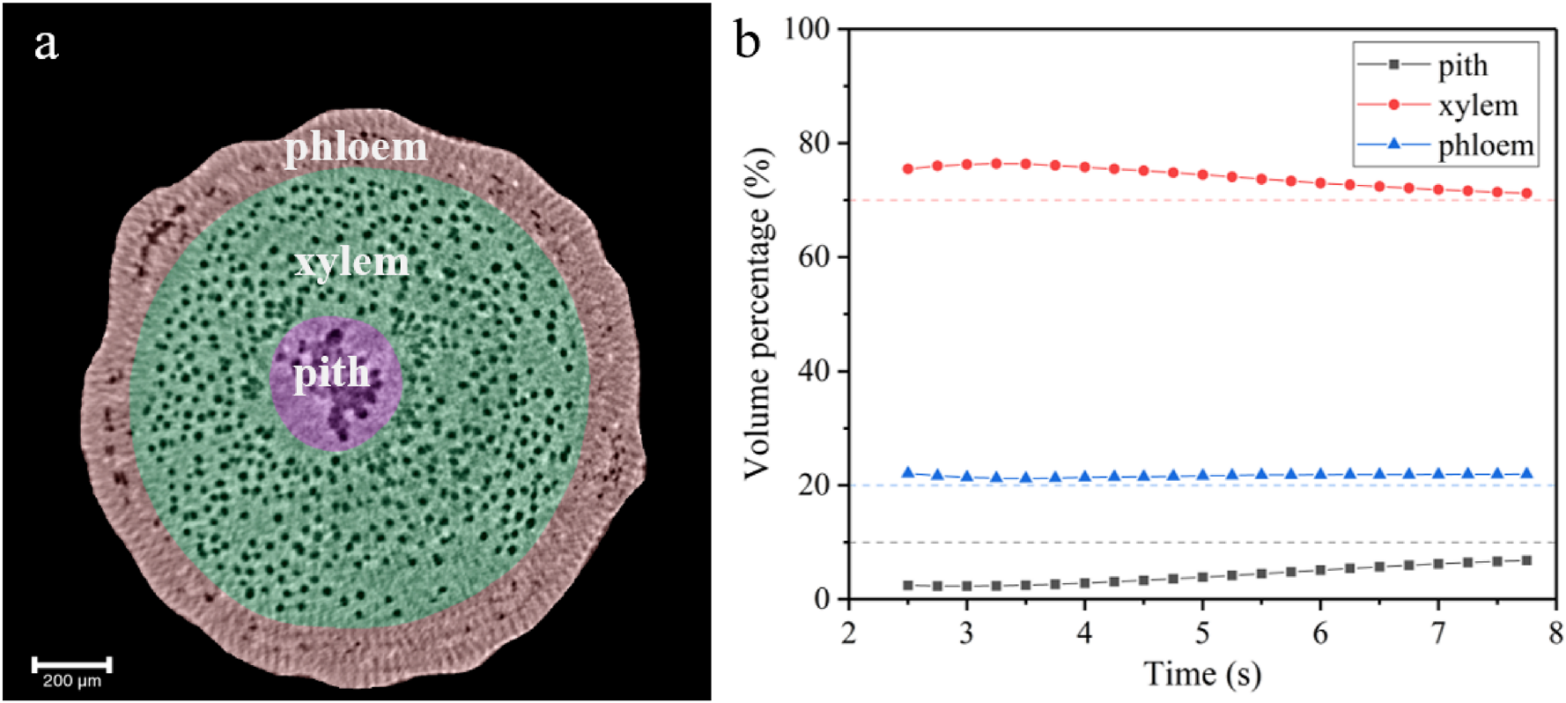
Different regions of willow branch and the proportion of their refilled water volume. (**a**) Phloem, xylem, and pith of the willow branch. (**b**) Evolution of the fraction of refilled water volume in the phloem, xylem and pith over time.

Similar to most woody plants, a willow branch is divided into three regions including phloem, xylem, and pith, as shown in Fig. 2a. Studies have shown that xylem is the region which has the greatest loss of hydraulic conductivity during drought^11,49^, therefore, most of the previous studies on plant water delivery focused on xylem^10^. According to the results given in the previous section, vessels play a key role in the process of water refilling. In addition, vessels are mainly concentrated in the xylem as shown in Fig. 1a, which infers that the xylem is an important region involved in water refilling. Results shown in Fig. 1c show that there are also a plenty of refilling signals in pith and phloem. Therefore, a quantitative analysis of the time evolution of the volume of refilled water in different regions, in particular the xylem, is crucial for understanding the hydrodynamics during the initial stage of cuttage.

According to the structural characteristics of phloem, xylem and pith, the three functional regions of the willow branch is parceled as shown in Fig. 2a. With this premise, we obtain the time evolution of the fraction of the refilled volume in different regions, as shown in Fig. 2b. In general, the proportion of the volume refilled by each region of the willow branch is relatively constant during the initial stage of cuttage. The contribution of pith is tiny at early stage, not exceeding 5%, and gradually increases with time but does not exceed 10%. The phloem contribution remains essentially constant throughout the refilling process, above 20%. The xylem contribution is 75% at early stage, then slowly decreases with time, but also remains above 70%. The results show that the xylem is the most important water refilling organ. As can be seen from Fig. 2a, the vessels are mainly distributed in the xylem. This implicates that vessel plays an important role in water refilling at the initial stage of willow cuttage (see Supplementary Fig. S4 online).

**Figure 3:**
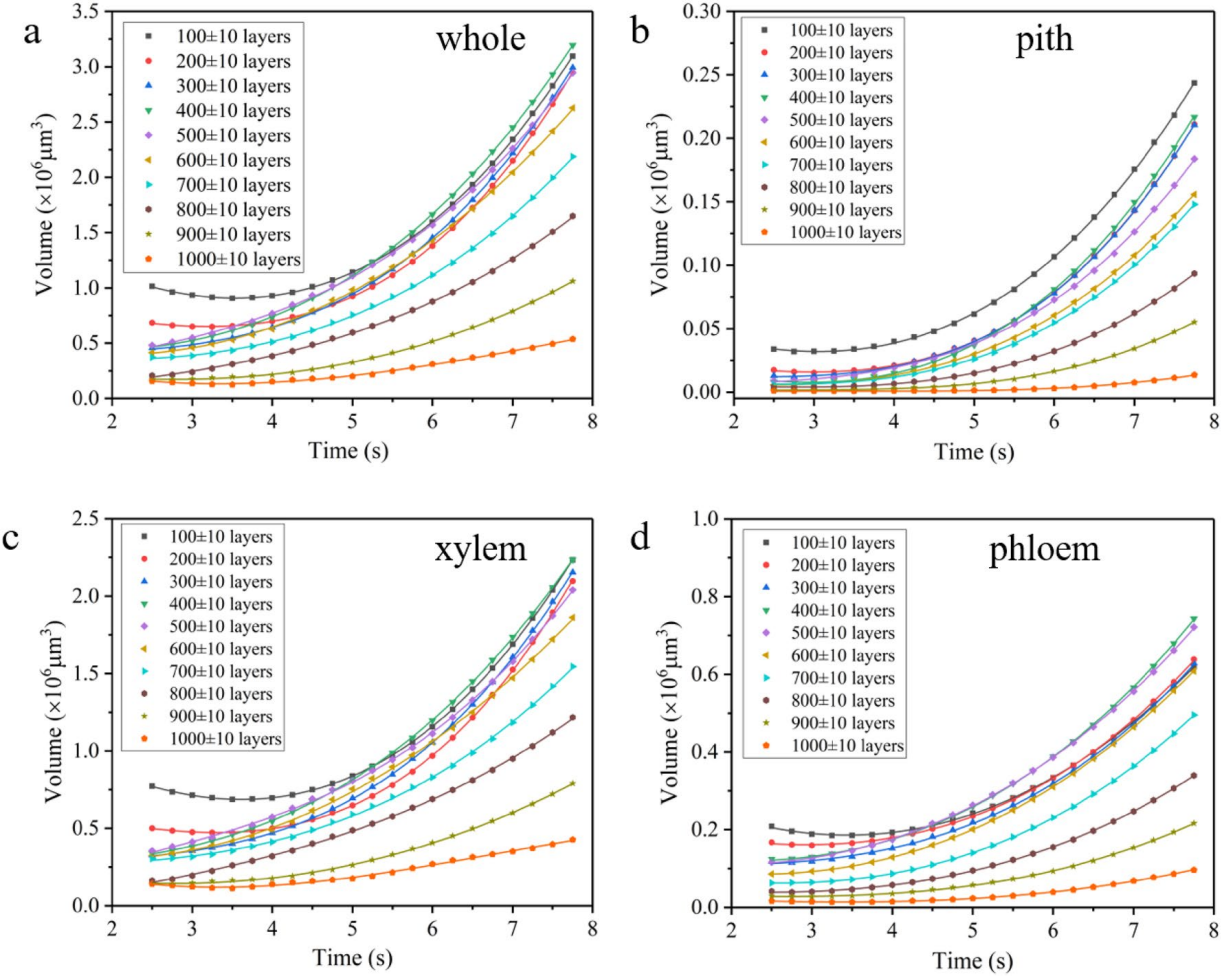
Evolution of the refilling volume at different cross section of whole willow branch (**a**), pith (**b**), xylem (**c**) and phloem (**d**) over time. (CT slice thickness 2.5 μm).

The sustainability of the water refilling during cuttage indicates that there is a driving force, so it is possible to obtain more information about the dynamics of the refilling by analyzing the water flow at different heights of the cutting. Firstly, to investigate the refilled flow at different heights, ± 10 layers of each 100 layers of the 1024 layers in the imaging field of view were taken as an analytical microelement with a thickness of 50 μm to quantitatively analyze the evolution of the refilled water volume within each microelement over time. The statistical results of each region are shown in Fig. 3, the refilled water volume of each layer gradually increases with time in general, and the closer to the water source, the larger increase trend. Consistent with the results in Fig. 2b, the xylem has the largest refilled water volume at each height of the xylem, and the pith has the smallest. From the perspective of position, the change rate of the refilled water volume with time before the 300 layers is relatively large, and then the change of each height of the refilled water volume tends to be monotonically uniform. From the perspective of the duration of refilling, the growth was relatively flat before 3.5 s, and the volume of each layer showed an obvious growth trend. This means that the refilling of water at the initial stage of cuttage requires a process of water pressure transfer and water infiltration and aggregation. The gradual decrease of water volume with height shows that there is also a pressure gradient at the beginning of cuttage.

### Mass force field driving water refilling

**Figure 4:**
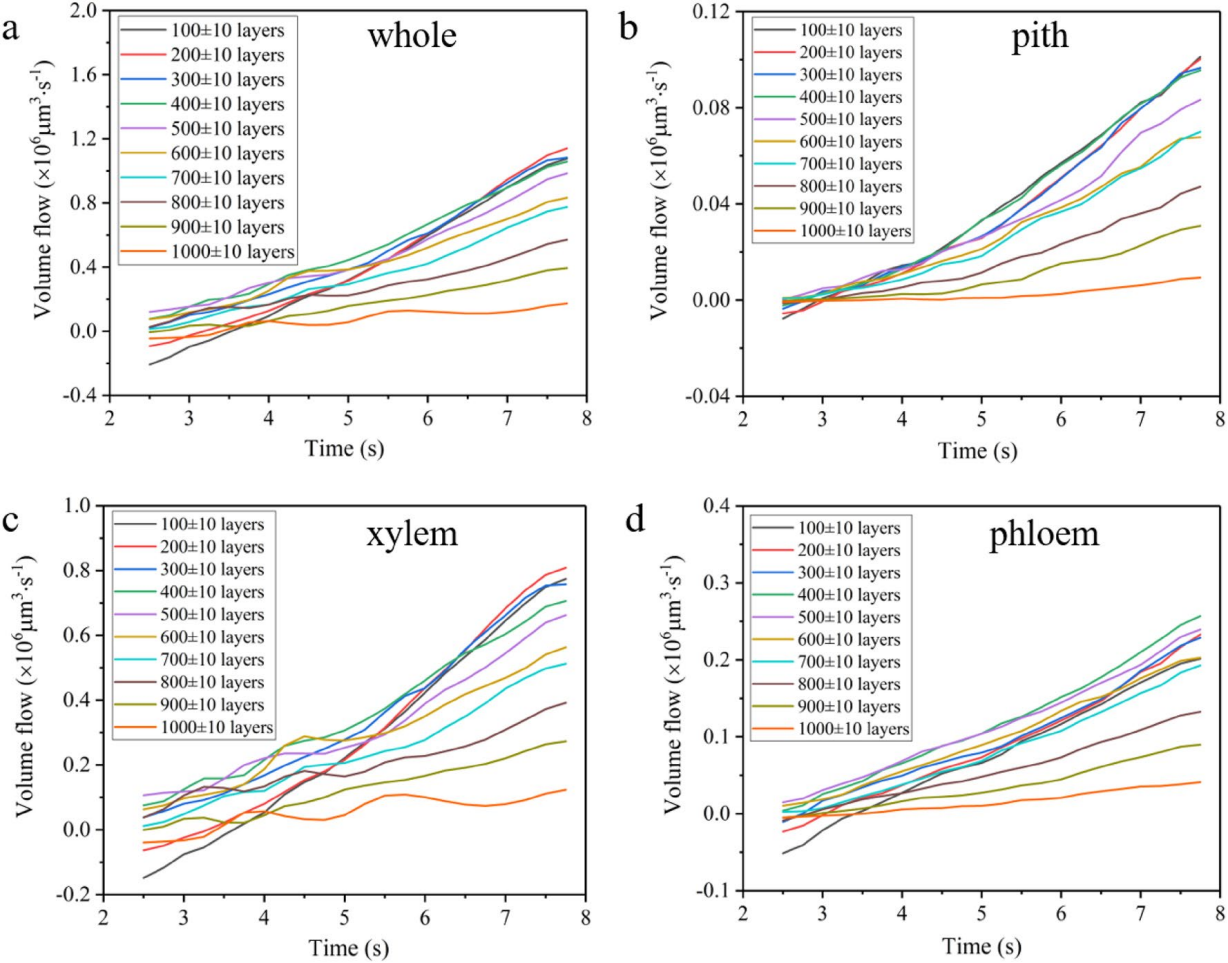
Evolution of the refilling flow of whole willow branch (**a**), pith (**b**), xylem (**c**) and phloem (**d**) over time.

Taking the first order differentiation of the refilled water volume-time curve in Fig. 3 can achieve the variation of water refilling flow over time. The results are given in Fig. 4, showing the curves of refilling flow at different regions and different positions of the willow branch over time. The refilled water flow increases quasi-linearly with time and the slope gradually decreases with positions from proximal to distal. Since the cross section of willow cutting is basically unchanged within the range of imaging field of view, it can be seen from the definition of flow that the flow rate of refilled water corresponds to the refilling speed one to one, and the change rate of flow over time corresponds to the change rate of velocity, that is, acceleration. As shown in Fig. 4, the flow rate of the refilled water at different heights is approximately constant. According to Newton’s Second Law, this means that the mass force driving the refilling at a given section is almost unchanged (see Supplementary the Definition of mass force online).

**Figure 5:**
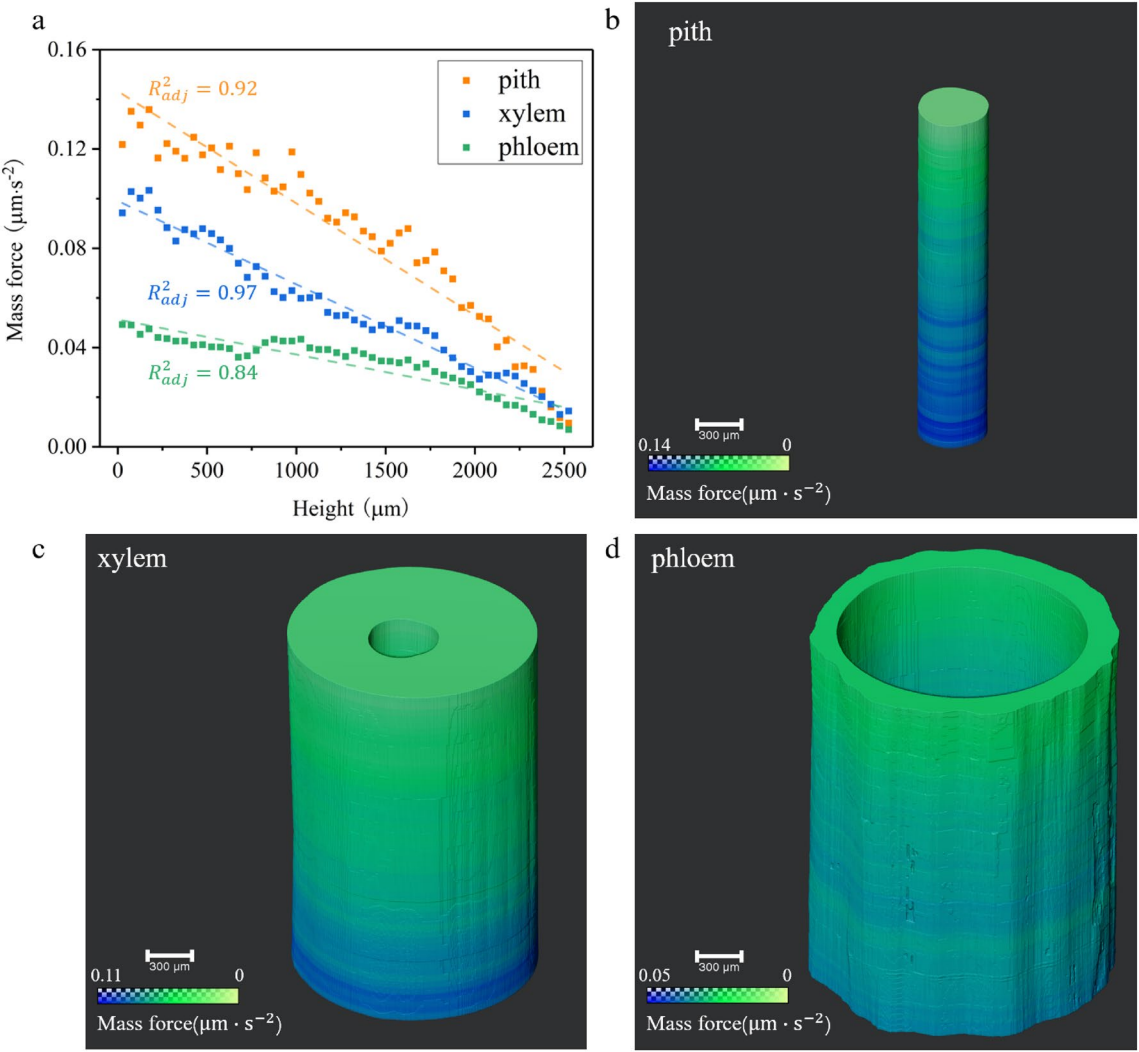
Spatial distribution of the mass force along different regions of the willow branch. (**a**) Scatter plots and linear fitting for the distribution of mass force in the pith, xylem and phloem of the willow branch. Panels (**b**)-(**d**), Three-dimensional spatial distribution of the mass force of pith (**b**), xylem (**c**), and phloem (**d**), the pseudo-color from blue to green indicates mass force from high to low.

According to the Supplementary equation (S9) online, the mass force represents the driving force per unit mass, which is numerically equal to the first-order time differential of the volume flow rate per unit area. A more precise differential element stratification method was used to statistically measure the flow rate of water at different heights and its evolution with time during refilling. The stratification is performed by treating every 20 slices as a unit, with no intervals between units. As shown in Fig. 5a, the distribution of the mass force along the height and its linear fit are plotted. The distribution of the mass force in pith, phloem, and xylem follows the similar gradient descent rules with different slopes. This indicates that the three functional regions are relatively independent in the refilling process. Their interfaces play a role in isolating the mass force. Although the previous analysis shows that the xylem contributes the largest amount of refilling, the mass force is not the largest because of its largest cross-sectional area. In addition, Fig. 5a shows that the pith with the smallest cross-sectional area is the region with the largest mass force, followed by the xylem, the phloem is the smallest. The dashed line in Fig. 5a gives the linear fit results for the mass force-height curves for each region of the willow branch. It shows slopes of − 4.51 × 10^−5^ s ^−2^, − 3.36 × 10^−5^ s^−2^, and − 1.41 × 10^−5^ s^−2^ for the pith, xylem, and phloem of the willow branch, respectively, with the slopes corresponding to the gradient of the mass force. Consequently, Fig. 5a indicates that there is a gradient descent of the mass force from proximal to distal at the initial stage of cuttage. The presence of this phenomenon is responsible for the occurrence and persistence of refilling. Therefore, the pith, i.e., the innermost region of the cutting, has the highest mass force, followed by xylem and the phloem. From inside to outside, the driving ability of the cutting is reduced successively, and the mass force of different regions is relatively independent, which is beneficial to the safety in remaining the refilling process to resist external interference. The phloem side is in direct contact with the atmosphere, itself isolating the effects of the atmosphere on the refilling inside the cutting, thus protecting the sustainability of the refilling ability of xylem, one of the organs contributing the most.

For a more intuitive study on the mass force in different regions of the willow branch, the pseudo-color 3D distribution of the mass force in pith (Fig. 5b), xylem (Fig. 5c), phloem (Fig. 5d), and whole (Supplementary Fig. S5b) were rendered. The change of the pseudo-color from blue to green indicates that the mass force gradually descents. In addition to the overall trend of the mass force varying from large to small with height, distinct circular bands are observed in all the three regions, which is consistent with the mass force fluctuation along the fitting straight line segment shown in Fig. 5a. From the result that the mass force is distributed in enhancement partially, it is found that there is a local accumulation process of the mass force at a specific location after conducting a certain distance to maintain the long-distance conduction of the refilling. After the mass force accumulates to a certain value, it continues to conduct backward. We called this phenomenon as an "inchworm effect". As shown in Fig. 5b–d, the dynamic range from the pith to the xylem to the phloem is 0.14, 0.10, 0.05, respectively, indicating that the distribution trend of the mass force in these three regions is roughly 3:2:1, decreasing from inside to outside. Despite the large difference in the mass force at the starting end, the mass force at 2500 μm tend to be consistent and close to 0. This means that in the 7.75 s period, the distribution range of the gradient-descent mass force is about 2.5 mm, and this gradient distribution continues to spread distally over time, achieving the refilling of the entire cutting.

## Discussion

Cuttage is a common plant cultivation method, and the key to its survival is the restoration of water refilling. Due to the need to satisfy simultaneously 3D microscopic spatial resolution, fast temporal resolution and high density resolution, traditional methods are difficult to be used for the in-situ study of water refilling in cuttage. We combined X-ray move contrast imaging with dynamic micro-CT to develop the MCXCT method, which enables high spatiotemporal resolution imaging of water refilling in willow branch at the initial stage of cuttage without resorting to contrast agent. Fused with the micro-CT structure of the cutting, the refilling 3D spatial position is further determined. Furthermore, the evolution of the refilled volume and flow rate over time can be quantitatively analyzed according to the MCXCT imaging results. This means that we can obtain the distribution of the unit mass force field driving the water refilling based on the relationship of refilling kinematics and dynamics, thus realizing the hydrodynamic analysis of the refilling process.

We report 3D dynamic live imaging of water refilling of cuttage without resorting to any contrast agent. In terms of the evolution of the refilled water volume over time, the proportion of refilling in the pith, xylem and phloem is roughly stable at the initial stage of cuttage. Xylem is the core region of refilling with a proportion of over 70%. Phloem is the key component of refilling, and its contribution is essentially constant over 20%. The contribution of pith is relatively small, not exceeding 10%. Hydrodynamics of the refilled water flow over time reveals the existence of a unit mass force field with a gradient along the refilling direction, which means that cutting plants also have a gradient force field to drive the recovery of water refilling, as predicted by Cohesion-Tension theory in normal plants. Since there is no tension generated by leaf transpiration in the cutting, moisture volatilization at the end of the cutting is considered to be responsible for the gradient mass force field. The results of the pith, xylem and phloem revealed that the mass force fields of refilled water in different functional regions are independently distributed and unconnected, which is beneficial to the security of water refilling. The decreasing mass force field distribution from inside to outside of the cutting can also minimize the interference of atmospheric pressure and external damage to refilling processes, which can further improve the refilling safety. Moreover, the "inchworm effect" of the mass force field indicates that the local enhancement of the mass force field occurs to accumulate power for subsequent refilling. Cutting realizes transfer of mass force in such a mild way to achieve a long-distance refilling of water. Lee et al. studied the refilling of *Phyllostachys bambusoides* leaves and a sprout using synchrotron X-ray phase contrast micro-imaging technique. They found that during refilling, the rising menisci in embolized vessels showed repetitive flow, i.e. they temporarily stopped at the end walls of the vessel elements while gas bubbles were removed. The meniscus then passed through the end wall at a faster rate than the speed of flow in the main vessels^50^. This is quite similar to the inchworm effect we reported in this paper.

Taking the cutting as a large straight-through tube, the refilling of the cutting does not fulfil the two basic assumptions of the CTT. However, if cutting is considered as a network of vessels, the short period of drying process before the cuttage is not sufficient to cavitate all the vessels within the cuttings. Theoretically, the CTT remains effective as long as there is at least one non-cavitated vessel. In fact, vessels are interconnected to form an intricate hydraulic network. As a result, after the brief drying process, there must be a number of tortuous hydraulic pathways within the cutting that connected the water source to the atmosphere at the top of the cutting and ensured hydraulic continuity within the cutting. In addition, the cutting was not sealed at the top of it, and the evaporation of water from the top of it can create tension. These two factors enable the refilling process within the cutting to partially comply with the CTT, causing the process to behave as driven by a gradient force field. However, our current research does not determine whether there are other driving factors for the refilling process of cuttage. Anyway, the findings presented in this paper is expected to help botanists to further understand the hydrodynamic mechanisms of water transport in plants.

## Methods

### Sample preparation of willow cutting

*Salix babylonica L.* is a typical species for propagation by cuttage, preferring light, warm, humid climates, with well-developed roots and rapid growth. Willow branch with the length of 10–12 cm and the diameter of about 2 mm was collected at the south gate of Shanghai Synchrotron Radiation Facility (31° 11′ 33″ N, 121° 35′ 34″ E) in early July as experimental samples in this study. Afterwards, place them in an indoor environment with a temperature of 23 °C and a humidity of 40% for about 0.5 h for cavitation treatment, and then conduct MCXCT experiments to insert willow branch into water to simulate the natural cuttage process. Due to the short duration of the experiment and the filter applied to the synchrotron radiation, no significant temperature increase or radiation damage was observed in the willow branch after the experiments. All methods were carried out in accordance with relevant guidelines.

### Experiments and image reconstruction

The MCXCT experiment was conducted on the X-ray imaging and biomedical application beamline BL13HB and X-ray optical test beamline BL09B of third-generation Shanghai Synchrotron Radiation Facility (SSRF) with storage ring energy of 3.5 GeV. The broadband white light mode was adopted to achieve high temporal resolution. The X-ray beam passing through the sample was converted by the scintillator (LuAG: Ce, 100 μm) into visible light, and then magnified by optical microscope with 8 × lens. The image was recorded by CMOS fast imaging detector (FASTCAM SA-Z, Photon), with the CMOS pixel size of 20 μm, bit depth of 12 bits, array size of 1024 × 1024 pixels. Therefore, the effective pixel size is 2.5 μm, which is sufficient to observe the refilling process in the willow branch.

During data acquisition, the rotation speed of the air-bearing stage was set to 240 rpm, with the exposure time for each frame of 33.7 µs, the frame rate of the detector of 1000 fps, and the exposure time of 7.75 s. This indicates that the sample rotated by 180° every 125 ms, and 125 projections were taken at the same time. A total of 62 sets of CT data were collected within 7.75 s. It is also necessary to consider the effect of centrifugal acceleration caused by rotation of the sample as it may interfere with experimental results. The formula for centrifugal acceleration of circular motion, a = ω^2^r, shows that the centrifugal acceleration is proportional to the radius. By substituting the relevant parameters into the equation, the centrifugal acceleration of the willow branch with a radius of 1 mm is less than 0.06 g (where g is the acceleration of gravitation). This means that at the rotation speed used in this experiment, the effect of the centrifugal acceleration on the natura refilling of the willow branch can be disregarded.

Firstly, the slice data of dynamic micro-CT was reconstructed based on the PITRE software^51^, and then the move contrast of the same slice reconstructed from different sets of CT data was reconstructed in chronological order. When the move contrast of the same slice was reconstructed, due to the constant density of water, the grayscale value fluctuations caused by the gas–water interfaces, similar to the form of the step-function signal, was utilized to realize the reconstruction of the trajectory of water refilling. The frequency of the water refilling signal is mostly concentrated in the low-frequency domain, while the noise during imaging is generally the high-frequency signal. Refilled water and complex backgrounds can be distinguished based on the principle of move contrast imaging, which greatly improves the imaging sensitivity of water refilling in the complex vessel-tissue network structure of the willow branch.

### Principle of MCXCT

Previous researches have been approved that move contrast X-ray imaging is highly sensitive for move signals in intricate system^43–45,47,48,52^. In order to obtain the 3D spatial position information of the refilling, we need to conduct the 3D reconstruction of the move contrast signal. In principle, extending MCXI to 3D imaging is logical. The challenge lies on data collection and image reconstruction. In 2D MCI, an image sequence can be acquired in a moderate exposure time and frame rate. In comparison, 3D move contrast imaging needs to tell the difference in a sequence of CT images. It means that move contrast X-ray micro-CT (MCXCT) needs higher photon flux, faster rotational speed, and faster frame rate for shooting. Meanwhile, projections at a fixed angle from different set of CT need to be aligned accurately. This is a great challenge for the synchronization of the detector with the rotating stage during the experiments. Usually, the mismatch of the projection images has to be less than one-half pixel, in order to reconstruct the 3D trajectory of water refilling along the vessels. In addition, sample need to be kept stable during high-speed rotating for dynamic data acquisition. The sample-instability-introduced misalignment is also required to be less than one-half pixel to avoid motion artifacts or erroneous signal during image reconstruction of MCXCT^48^. As for image reconstruction of MCXCT, we need to reconstruct the three-dimensional conventional contrast microstructure of the cutting and also the move contrast trajectory of water refilling respectively. The occurrence of water refilling was then localized by image fusion of the two sets of CT in phase contrast and move contrast.

### Supplementary Information

Supplementary Information 1.

Supplementary Video 1.

## Data Availability

The data that support the findings of this study are available from the corresponding author upon reasonable request.
