# Supplementary material for: Measurement of mass force field driving water refilling of cuttage
Source: Sci Rep. 2024 Apr 18;14:8947. doi: 10.1038/s41598-024-59716-x (PMC11026483; doi:10.1038/s41598-024-59716-x)
Supplement: Supplementary file 1 — Supplementary Information 1. [file 41598_2024_59716_MOESM1_ESM.docx]

**Measurement of mass force field driving water refilling of cuttage**

Mingwei Xu^1,2,3†^, Ke Li^1†^, Yanling Xue^1^, Feixiang Wang^1^, Zhixuan Liu^4^, Tiqiao Xiao^1,2,3*^

^1^ Research Center for Shanghai Synchrotron Radiation Facility, Shanghai Advanced Research Institute, Chinese Academy of Sciences, Shanghai 201204, China
^2^ Shanghai Institute of Applied Physics, Chinese Academy of Sciences, Shanghai 201800, China

^3^University of Chinese Academy of Sciences, Beijing 100049, China

^4^Hunan Rice Research Institute, Hunan Academy of Agricultural Sciences, Changsha 410125, China

† These authors contributed equally.

* Corresponding author: tqxiao@sari.ac.cn.

# Supplementary Information

**1 Diagrams of the experimental setup and micrographs of the willow branch cross section.**

Before the experiment, four layers of 15 μm thick aluminum foils were placed in the optical path in front of the sample to absorb low-energy X-rays, in order to eliminate the effect of radiation damage. The experimental device is shown in Fig. S1. Willow branch was fixed on the polymethyl methacrylate (PMMA) holder with a water tank at the bottom through adhesive tape. A round hole is opened on the upper surface of the water tank to facilitate insertion of the willow branch. The α-cyanoacrylate glue/adhesive (502 glue) was used to fix the holder on the air-bearing stage to keep the sample stable during the rotary scanning process. Before the experiment, the water tank was kept dry. When everything is ready, the water tank was filled with water through a medical syringe, then the photon shutter was opened, and shooting started immediately after the rotation speed of the air-bearing stage was stable. Due to the safety interlock setting of the experimental hutch, the photon shutter was opened 10-15 s after water injection, and data acquisition was started. The detector was placed 53 cm away from the sample to ensure sufficient phase propagation distance and the contrast of the gas-water interfaces were further enhanced. As shown in Fig. S1a, the imaging area is marked with a red rectangle.

The sample holder is made of PMMA with high integrity and low scattering to X-rays, therefore, the stability of the sample is ensured during rotation, and the impact on the projection image is minimized. The sample base is a cylindrical sealed water tank with an inner diameter of 68 mm, a depth of 5 mm and a volume of 18 ml. During the experiment, a medical syringe is used to fill the injection hole shown in Fig. S1b until the water tank is full.


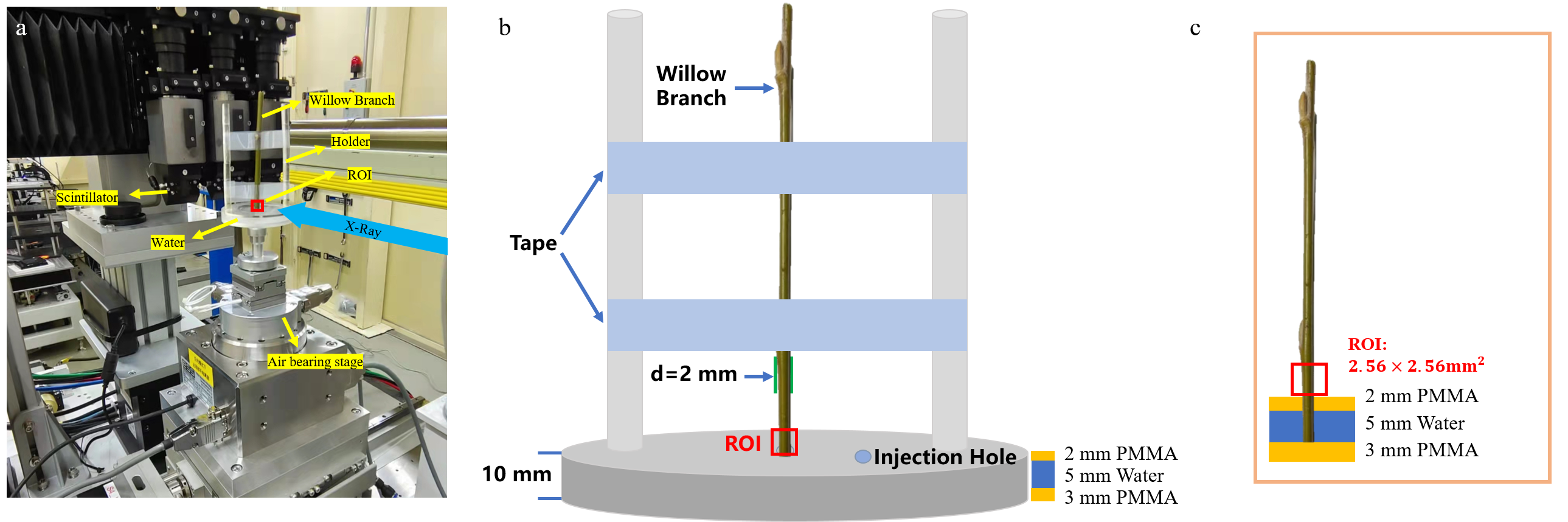


**Figure S1.** Schematic of the experimental setup of MCXCT. (**a**) Experimental setup. (**b**) Schematic diagram of a willow branch and its holder. (**c**) The location of the ROI (Region of Interest) of the willow branch with respect to the water tank.


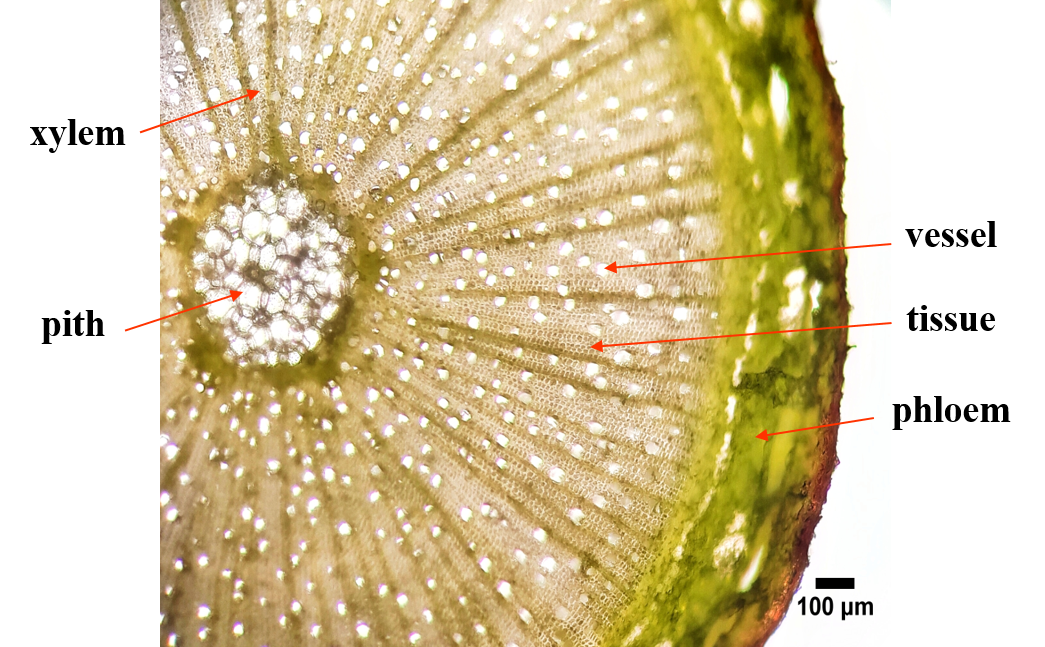


**Figure S2.** Micrograph of cross section of a willow branch. Complex tissue, vascular bundle network structure and phloem, xylem and pith components can be observed.

**2 Principle of move contrast X-ray imaging**

Move modes of various components of the sample will have different modulation effects on the incident X-ray. The time-domain fluctuation *g* (*x*, *y*, *t*) of the grayscale value at a point (*x*, *y*) in the modulated emergent light field reflects the move characteristics of the corresponding point in the sample. In the time domain, the *g* (*x*, *y*, *t*) of the grayscale fluctuation caused by component movement is usually weak, and tends to be submerged by background noises. This means that it is hard to achieve sufficient contrast in the real space. However, the frequency of the sample to the light field modulation is generally quite different, and the contrast fluctuation of the move components in the frequency domain can be used for imaging. The spectral representation of the point (*x*, *y*) can be obtained by converting the discrete Fourier transform *g* (*x*, *y*, *t*) to the frequency domain:

|  | $G\left(x,y,k\right)=\sum_{t=0}^{N-1} g(x,y,t)\mathrm{e}^{-j\frac{2\pi}{N}kt}$, | (S1) |
| --- | --- | --- |

Where *G* (*x*, *y*, *k*) represents the spectrum of *g* (*x*, *y*, *t*), and *N* refers to the sequence length. The spectrum form expressed by trigonometric function is obtained by expanding equation (S1) through Euler formula:

|  | $G\left(x,y,k\right)=\sum_{t=0}^{N-1} g(\mathrm{x},\mathrm{y},\mathrm{t})\cdot\left[\cos\left(\frac{2\pi kt}{N}\right)-j\sin\left(\frac{2\pi kt}{N}\right)\right]$, | (S2) |
| --- | --- | --- |

The amplitude and phase of the spectrum are as follows:

|  | $A(x,y,k)=\sqrt{\mathrm{Re}^{2}(G(x,y,k))+\mathrm{Im}^{2}(G(x,y,k))}$, | (S3) |
| --- | --- | --- |
|  | , | (S4) |

Where the amplitude represents the fit of the sample’s move mode to the target move mode, that is, the trajectory of the move signal. In this paper, it represents the trajectory of water. The phase value represents the time information of the move signal, corresponding to the time when the refilling occurs. According to the linear property of Fourier transform, the spectrum of move contrast’s amplitude information can be divided into combinations of different frequencies, and the move track information of the interesting signals can be obtained by selecting the appropriate spectrum range ^1^. Equation (S3) can be transformed into:

|  | $A(x,y)=A(x,y,0)+\sum_{k=1}^{k_{LP}-1} A(x,y,k)+\sum_{k=k_{LP}}^{k_{HP}} A(x,y,k)+\sum_{k=k_{HP}+1}^{N/2} A(x,y,k)$, | (S5) |
| --- | --- | --- |

Where *A* (*x*, *y*, *0*) refers to the average strength of the signal, that is, the DC component of the spectrum. $\sum_{k=1}^{k_{LP}-1} A(x,y,k)$, $\sum_{k=k_{LP}}^{k_{HP}} A(x,y,k)$ and $\sum_{k=k_{HP}+1}^{N/2} A(x,y,k)$ represent the low-frequency component, band-pass component and high-pass component of the signal, respectively; *k_LP_* and *k_HP_* represent the spectral range of the band-pass filter, respectively. The X-ray image’s grayscale value change caused by water infiltration or filling during the refilling process is usually represented as a low-frequency signal. The appropriate spectrum range and band-pass filtering are used to distinguish the refilling trajectory signal from the complex background noise and high-frequency noise, so as to realize the highly sensitive imaging of tissue infiltration and vessel delivery.

To obtain the time of refilling, the phase information of move contrast imaging is used. It is assumed that the target moves from the space-time position (*x_0_*, *y_0_*, *t_0_*) to the adjacent space-time position (*x_1_*, *y_1_*, *t_0_*+Δ*t*) within Δ*t* time. Due to the short distance, the pixel grayscale value *g* (*x_0_*, *y_0_*, *t_0_*) can be considered to be approximately unchanged. According to equation (S2) and equation (S4), the spectrum of the position (*x_1_*, *y_1_*, *t_0_*+Δ*t*) can be changed to ^1^:

|  | $\begin{matrix} G\left(x_{1},y_{1},k\right)\approx\sum_{t=0}^{N-1} g(\mathrm{x}_{1},\mathrm{y}_{1},\mathrm{t}_{0}\text{+}\Delta\text{t})\mathrm{e}^{-j\frac{2\pi}{N}k\left(\mathrm{t}_{0}\text{+}\Delta\text{t}\right)} \\ \text{=G}\left(x_{0},y_{0},k\right)\mathrm{e}^{-j\frac{2\pi}{N}k\Delta t} \end{matrix}$, | (S6) |
| --- | --- | --- |

This means that a phase shift factor $\mathrm{e}^{-j\frac{2\pi}{N}k\Delta t}$ is introduced into the spectrum signal, and the time change is characterized by the phase shift of the move contrast. The trajectory of refilling is obtained based on the amplitude information of move contrast, and the time of refilling is obtained based on its phase information, so that the spatial position and time node information of water in the refilling process can be obtained simultaneously by using move contrast imaging.

**3 Temporal and spatial distribution of MCXCT refilling (2D/3D)**


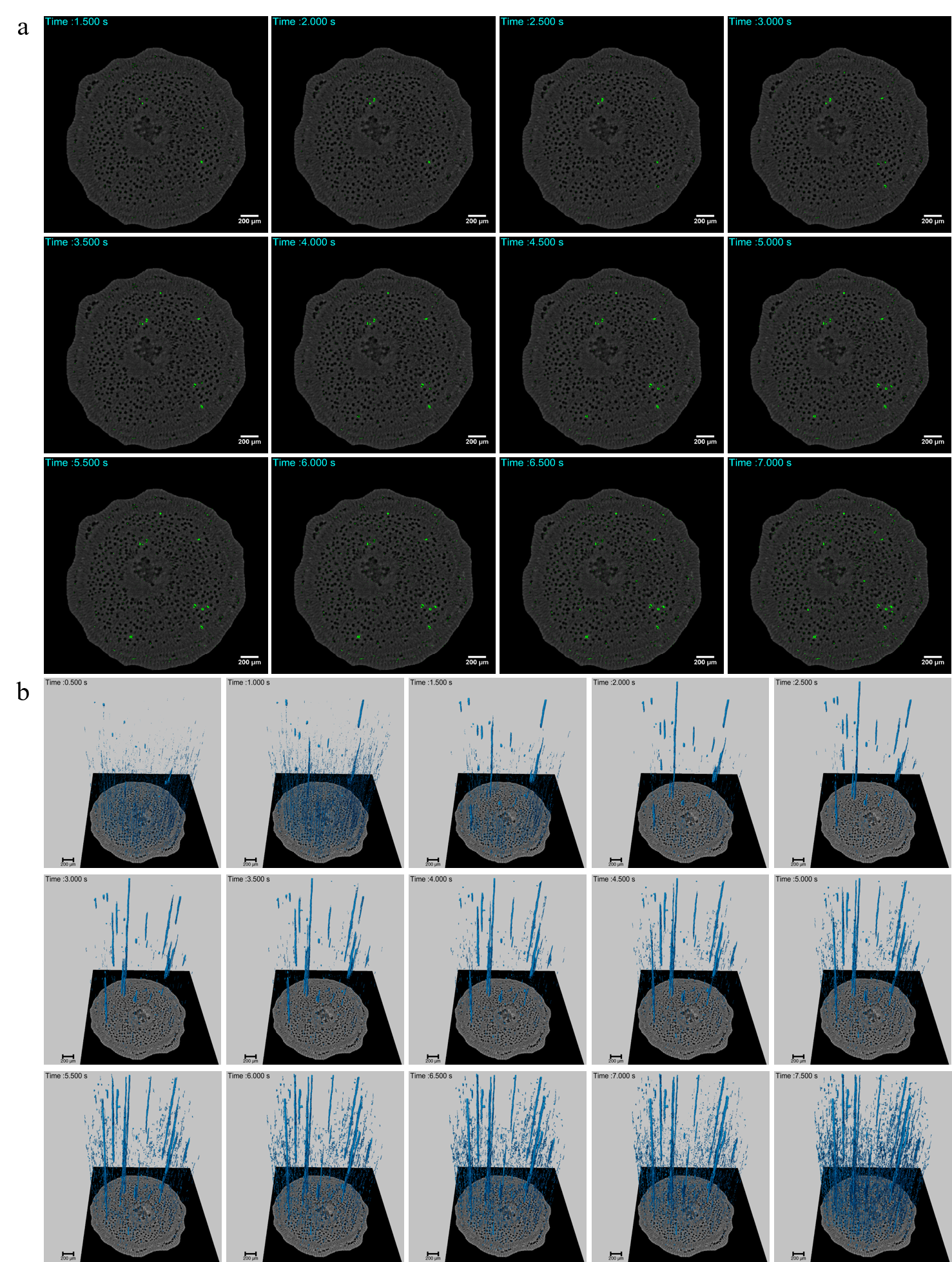


**Figure S3.** Time evolution of willow branch refilling in slice No. 500 (**a**) and three-dimensional space (**b**).

**4 Spatial structure of willow branch vessels and tissues**


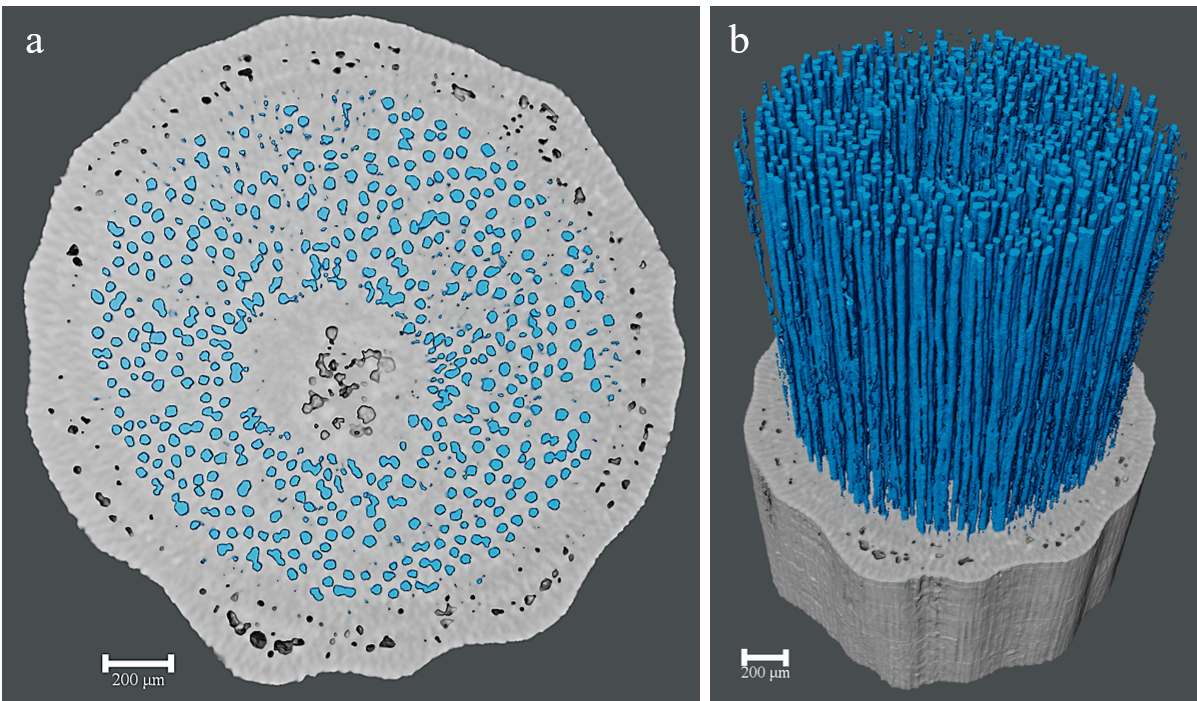


**Figure S4.** Distribution of vessels in the slice No. 500 (**a**) and three-dimensional space (**b**) of willow branch. The gray elements represent tissues, and the blue elements represent vessels.

**5 Definition of mass force**

Rate of flow refers to the amount of fluid flowing through the effective section of the closed pipe in per unit time. When the amount of fluid is expressed in volume, it is called volume flow, usually denoted by *Q*. Macroscopically, the willow branch can be seen as a closed flow pipe during the refilling process. *s* refers to the cross-sectional area of the willow branch, so the average flow velocity $\overline{v}$ of the refilling water can be expressed as:

|  | $\overline{v}=\frac{Q}{s}$ | (S7) |
| --- | --- | --- |

There is no significant change in the sample size during the experiment, hence *s* is constant. According to Newton's second law ^2^, acceleration *a* refers to the force exerted on an object per unit mass:

|  | $a=\frac{d\overline{v}}{dt}=\frac{F}{m}$ | (S8) |
| --- | --- | --- |

The mass force *F_m_* refers to the driving force per unit mass ^3^, i.e., the force pulling the unit mass of water to accomplish the refilling process, which is similar to the well-known acceleration. Substituting equation (S7) into equation (S8), the mass force is obtained, as follows:

|  | $F_{m}=\frac{F}{m}=\frac{1}{s}\cdot\frac{dQ}{dt}$ | (S9) |
| --- | --- | --- |

which is numerically equal to the first-order time differential of the volume flow rate per unit area.

**6 Mass force field of the whole willow branch**


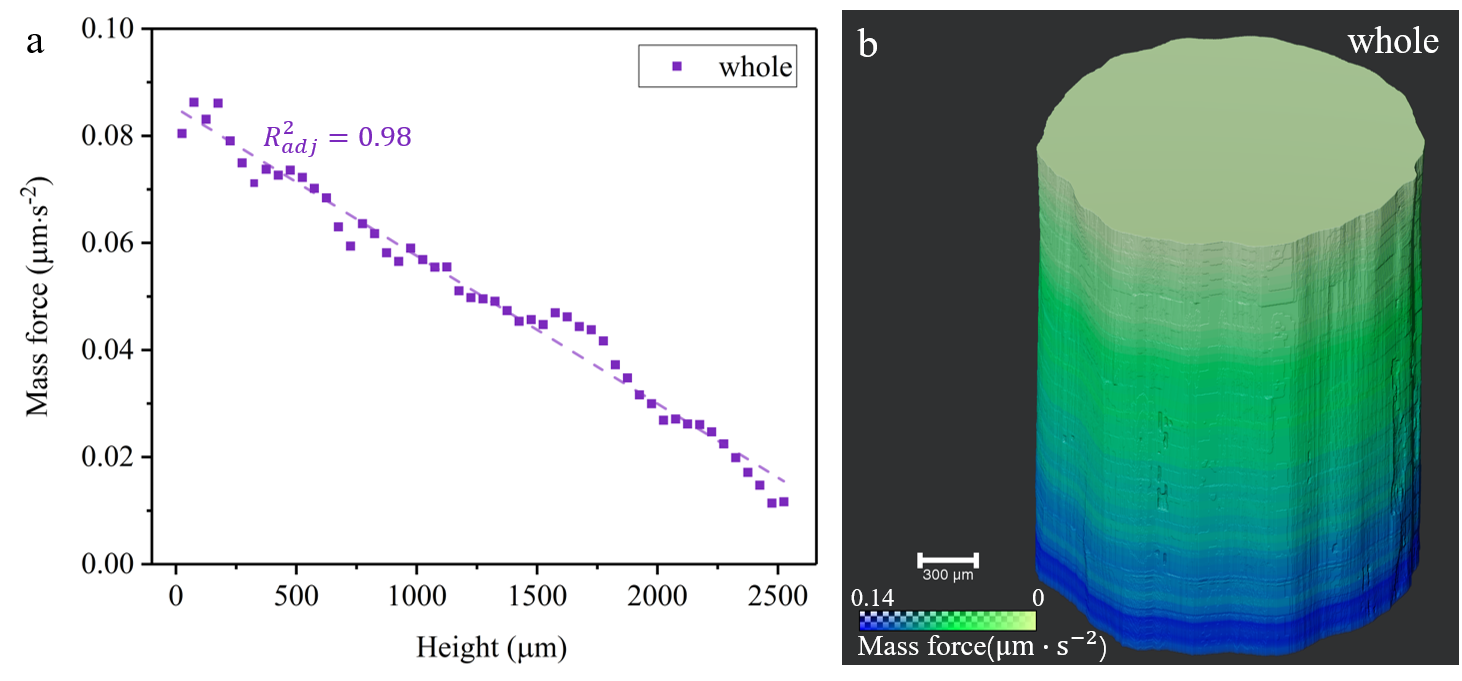


**Figure S5.** Mass force field of the whole willow branch. (**a**) Scatter plots and linear fitting for the mass force. (**b**) Pseudo-color three-dimensional spatial distribution of the mass force, the pseudo-color from blue to green indicates mass force from high to low.

# References

1. Li K. *X-ray imaging to complex systems and its application in low-Z materials*, University of Chinese Academy of Sciences, (2021).

2. Newton, I. *Philosophiae naturalis principia mathematica*. Vol. 1 (G. Brookman, 1833).

3. Shang DY & Zhong LC. *Heat transfer of laminar mixed convection of liquid*. (Springer, 2016).
